# Capillary networks and follicular marginal zones in human spleens. Three-dimensional models based on immunostained serial sections

**DOI:** 10.1371/journal.pone.0191019

**Published:** 2018-02-08

**Authors:** Birte S. Steiniger, Christine Ulrich, Moritz Berthold, Michael Guthe, Oleg Lobachev

**Affiliations:** 1 Institute of Anatomy and Cell Biology, University of Marburg, Marburg, Germany; 2 Institute of Psychology, University of Marburg, Marburg, Germany; 3 Institute of Computer Sciences, University of Bayreuth, Bayreuth, Germany; University of Miami, UNITED STATES

## Abstract

We have reconstructed small parts of capillary networks in the human splenic white pulp using serial sections immunostained for CD34 alone or for CD34 and CD271. The three-dimensional (3D) models show three types of interconnected networks: a network with very few long capillaries inside the white pulp originating from central arteries, a denser network surrounding follicles plus periarterial T-cell regions and a network in the red pulp. Capillaries of the perifollicular network and the red pulp network have open ends. Perifollicular capillaries form an arrangement similar to a basketball net located in the outer marginal zone. The marginal zone is defined by MAdCAM-1^+^ marginal reticular stromal cells. Perifollicular capillaries are connected to red pulp capillaries surrounded by CD271^+^ stromal capillary sheath cells. The scarcity of capillaries inside the splenic white pulp is astonishing, as non-polarised germinal centres with proliferating B-cells occur in adult human spleens. We suggest that specialized stromal marginal reticular cells form a barrier inside the splenic marginal zone, which together with the scarcity of capillaries guarantees the maintenance of gradients necessary for positioning of migratory B- and T-lymphocytes in the human splenic white pulp.

## 1 Introduction

The spleen is the organ with the most enigmatic microvasculature, both in humans and in rats or mice [1–3]. In all three species the main part of the organ, the so-termed red pulp, harbours two types of microvessels, namely capillaries and sinuses, which form the end of the arterial tree and, respectively, the beginning of the venous part of the circulation. The exceptional feature is that both types of microvessels are not connected to one another in humans [4]. The capillaries have open ends delivering the blood to strands of reticular connective tissue. Blood plasma and all blood cells finally enter venous sinuses from the outside via openings in their walls. Thus, the spleen is the only organ exhibiting an open circulation where blood flows in spaces not delimited by endothelial cells, but by fibroblasts. Apart from different types of fibroblasts, specialised macrophages, plasma cells and potentially also mast cells form more sessile cell populations in the splenic red pulp.

Besides this very special structure of the red pulp, the spleen also hosts dense accumulations of more or less migratory lymphocytes, which are named the white pulp. The white pulp is populated by T- and B-lymphocytes, which migrate in special compartments located in the vicinity of branches of the splenic artery termed central arteries. Details of splenic white pulp compartments were first described in rats and mice [3,5,6]. The white pulp consists of periarterial lymphatic sheaths (PALSs) predominantly occupied by T-lymphocytes and of hemispherical follicles attached to the sheaths where B-lymphocytes predominate. Both lymphocyte types are attracted by compartment-specific resident connective tissue cells termed fibroblastic reticulum cells (FRCs) in case of PALSs and follicular dendritic cells (FDCs) in case of follicles. During B-cell immune reactions secondary follicles are formed, which comprise a germinal center with proliferating antigen-stimulated B-lymphocytes and a surrounding mantle zone formed by naive recirculating B-lymphocytes. An additional compartment is the marginal zone (MZ), which surrounds both PALSs and follicles in rats and mice. The MZ is separated from these compartments by a blood-filled cleft called the marginal sinus and by marginal metallophilic macrophages (MMMs, [7,8]). The rat MZ is difficult to classify because it belongs to the open circulation system and harbours a minor number of red blood cells, but at the same time it is densely populated by MZ B-lymphocytes. Thus, it may be regarded as part of the red pulp or of the white pulp. In rats and mice B-lymphocytes in the MZ are a heterogeneous population comprising memory B-cells [9,10], but also B-lymphocytes of an independent MZ developmental pathway [11].

In adult human spleens T- and B-cell areas of the white pulp and also the MZ are arranged differently from rats and mice [12–15]. In contrast to both rodent species, the PALSs are of limited length and spherical follicles form the major compartment of the white pulp. Human CD27^+^ memory B-cells [16] are only present at the surface of follicles and are absent from the surface of the PALSs. A marginal sinus and MMMs are also lacking in humans. The human MZ cannot be defined by B-cells, because CD27^+^ memory B cells both occur among the most superficial FDCs in the follicular mantle zone and among marginal reticular cells (MRCs, [15]). These cells form a third stromal cell type superficial to FRCs and FDCs. They have been first described in mouse lymph nodes [17]. It is not clear, whether the superficial follicular stromal cells in human spleens are totally identical to mouse MRCs. MRCs are the only organ-specific cells defining a MZ-equivalent in the human spleen.

In previous publications we have analysed the composition of an additional species-specific trait of human spleens, the capillary sheaths [18]. The most unusual cell type in these structures are strongly CD271 positive (CD271^++^) capillary sheath cells. These cells represent the most strongly CD271^+^ cell type in human spleens. CD271 is also present in FDCs, FRCs and with less intensity in ubiquitous splenic fibroblasts [14]. It is highly likely that sheath cells represent the only sessile cells in human capillary sheaths. They are surrounded by macrophages and recirculating B-cells [18]. Capillary sheaths are absent in mouse or rat spleens. This is exceptional, because capillary sheaths or equivalent structures termed ellipsoids occur in most vertebrate species.

The microvasculature of human spleens has been investigated in 3D before [1, 4, 19]. However, up to now the exact position of splenic capillaries in the white pulp has not been localised. To obtain a more comprehensive overview of all capillaries in a human spleen, we now reconstructed serial sections stained for CD34 alone and for CD34 followed by visualisation of CD271. We describe three different interconnected capillary networks. In addition, we definitively prove that humans do not exhibit a marginal sinus. The 3D models developed are quality controlled by direct comparison to the original immunostained serial sections in virtual reality (VR).

## 2. Results

### 2.1 Stromal cells, B lymphocytes and microvessels at the surface of splenic follicles

**Fig 1:**
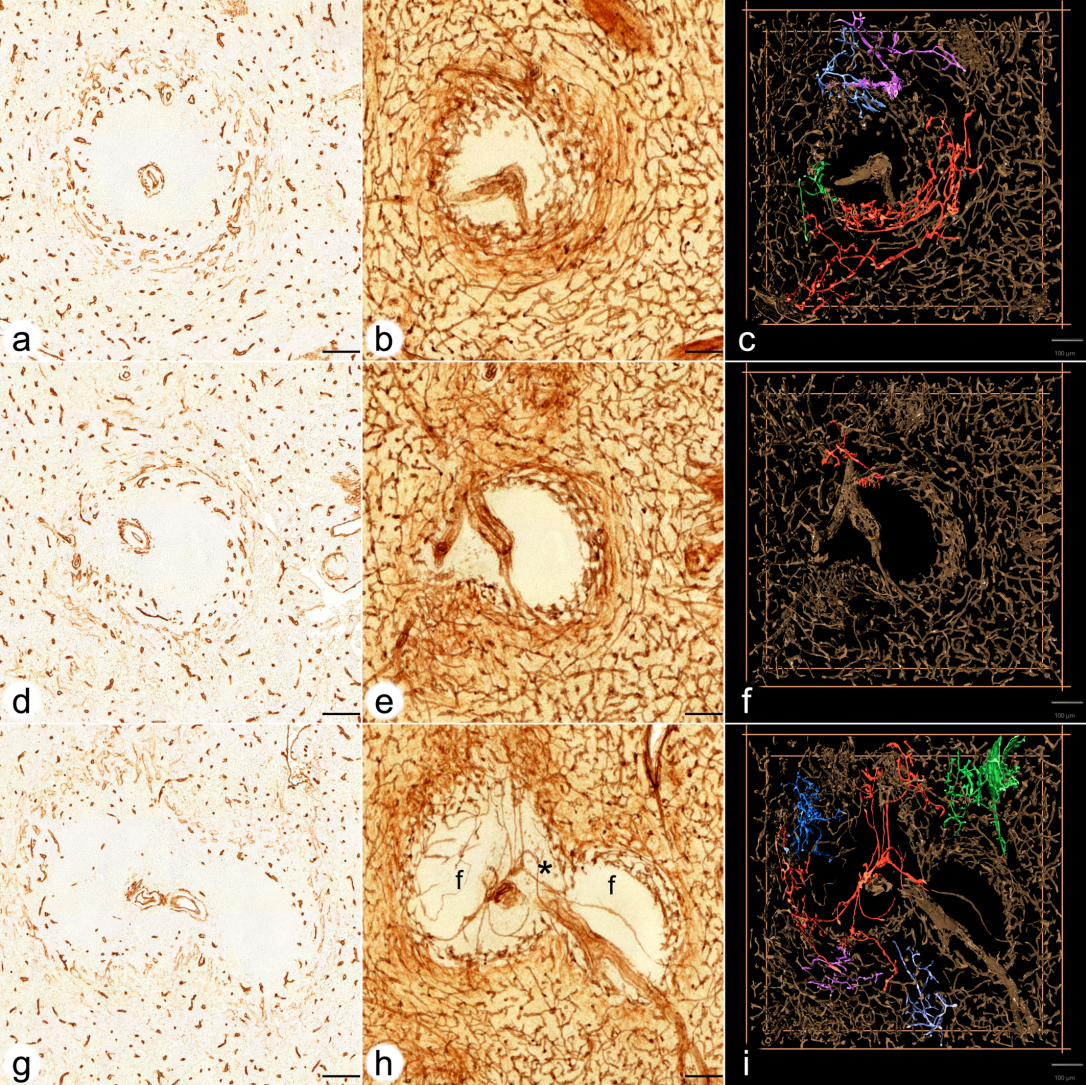
Visualisation of perifollicular, red pulp and white pulp capillaries centered on three different follicles. **(a,b,c) ROI 1 (a)** first section stained for CD34, **(b)** overlay of all 24 registered sections, **(c)** first frame of video showing a 3D model with four connections among perifollicular and red pulp capillaries highlighted in different colours. **(d,e,f) ROI 2 (d)** first section stained for CD34, **(e)** overlay of all 24 registered sections, **(f)** first frame of video showing a 3D model with one connection among perifollicular and red pulp capillaries highlighted in red colour. **(g,h,i) ROI 3 (g)** first section stained for CD34, **(h)** overlay of all 24 registered sections, **(i)** first frame of video showing a 3D model with five connections among perifollicular and red pulp capillaries highlighted in different colours. (g,h,i) show two follicles (f in h) and a part of a PALS (asterisk in h) in between. Internal capillaries arise from a central artery in the PALS. Staining of red pulp sinuses has been reduced as documented in suppl. Fig 2 in c,f,i. CD34 is present in capillary endothelia, adventitial fibroblasts of arteries, perifollicular sinus endothelia (weak) and in fibroblasts in trabeculae. All scale bars = 100 μm.

**Fig 2:**
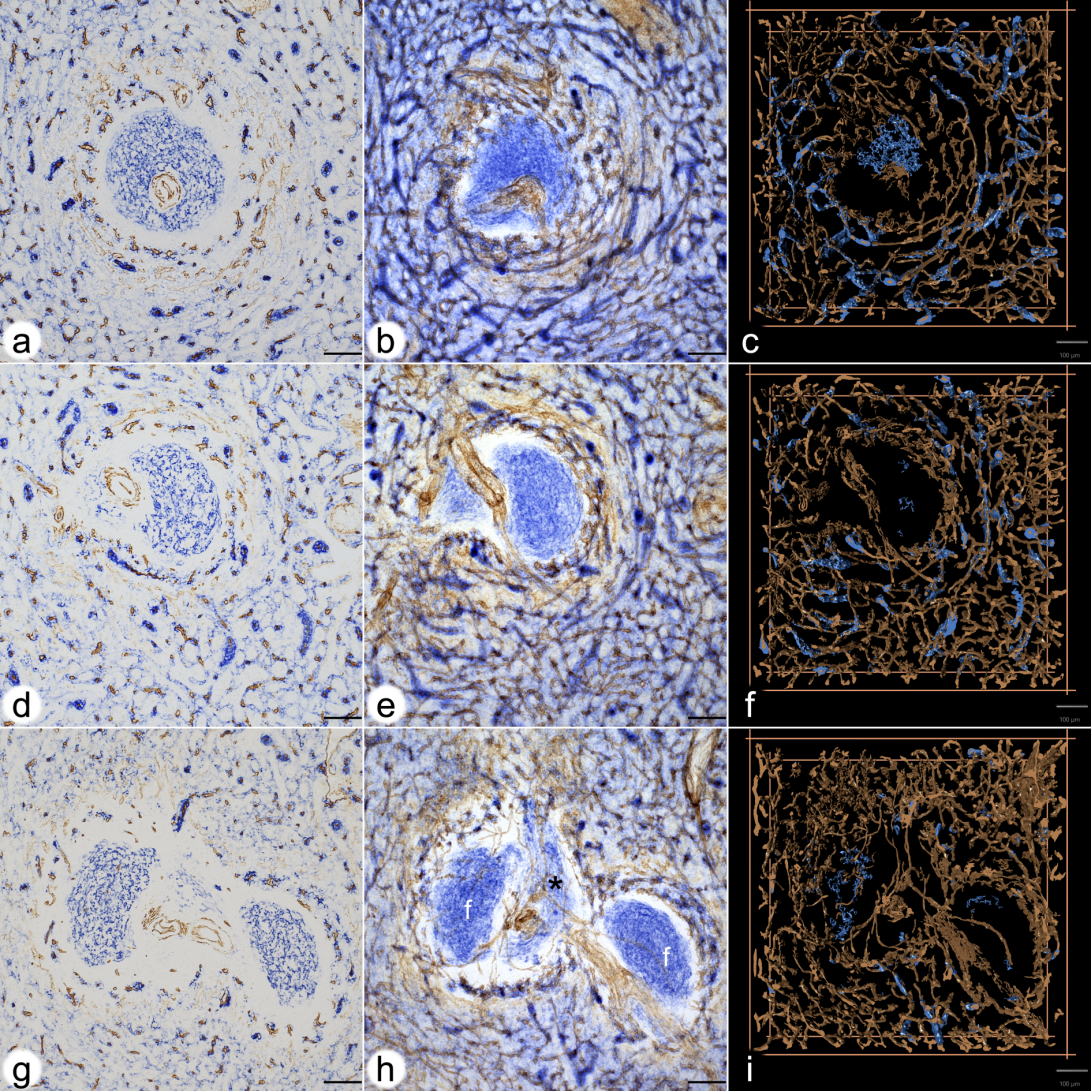
Visualisation of CD271^+^ sheaths around red pulp capillaries connected to the perifollicular network of the three follicles. **(a,b,c) ROI 1(a)** first section stained for CD34 (brown) and for CD 271 (blue), **(b)** overlay of all 24 registered sections, **(c)** first frame of video of the 3D model**(d,e,f) ROI 2(d)** first section stained for CD34 (brown) and for CD 271 (blue), **(e)** overlay of all 24 registered sections, **(f)** first frame of video of the 3D model**(g,h,i) ROI 3(g)** first section stained for CD34 (brown) and for CD 271 (blue), **(h)** overlay of all 24 registered sections, **(i)** first frame of video of the 3D modelCD271 is most strongly expressed in stromal capillary sheath cells. FDCs in follicles, FRCs in a PALS and ubiquitous interstitial fibroblasts are also positive. Staining of red pulp sinuses for CD34 and of ubiquitous fibroblasts for CD271 has been reduced by choosing an appropriate iso-value.(g), (h) and (i) show two follicles (f in h) and a part of a PALS in between. Internal capillaries arise from a central artery in the PALS (asterisk in h).All scale bars = 100 μm.

The distribution of CD34, CD271, CD27 and other moleculesin human spleens has already been described by us in 2D [12–15, 20], but in the present study some additional details were found. CD34 is present in endothelial cells of larger splenic arterial and venous vessels and in capillaries. In addition, different types of fibroblasts located periarterially, within trabeculae and in the splenic capsule are stained. Perifollicular sinus endothelia are also faintly positive, while the majority of the splenic red pulp sinuses appear to be CD34 negative (Fig 1A, 1B, 1D, 1E, 1G and 1H, Fig 2A, 2B, 2D, 2E, 2G and 2H). Single round CD34^+^ cells are also present in the red pulp, which may represent haematopoietic precursor cells in the open circulation.

**Fig 3:**
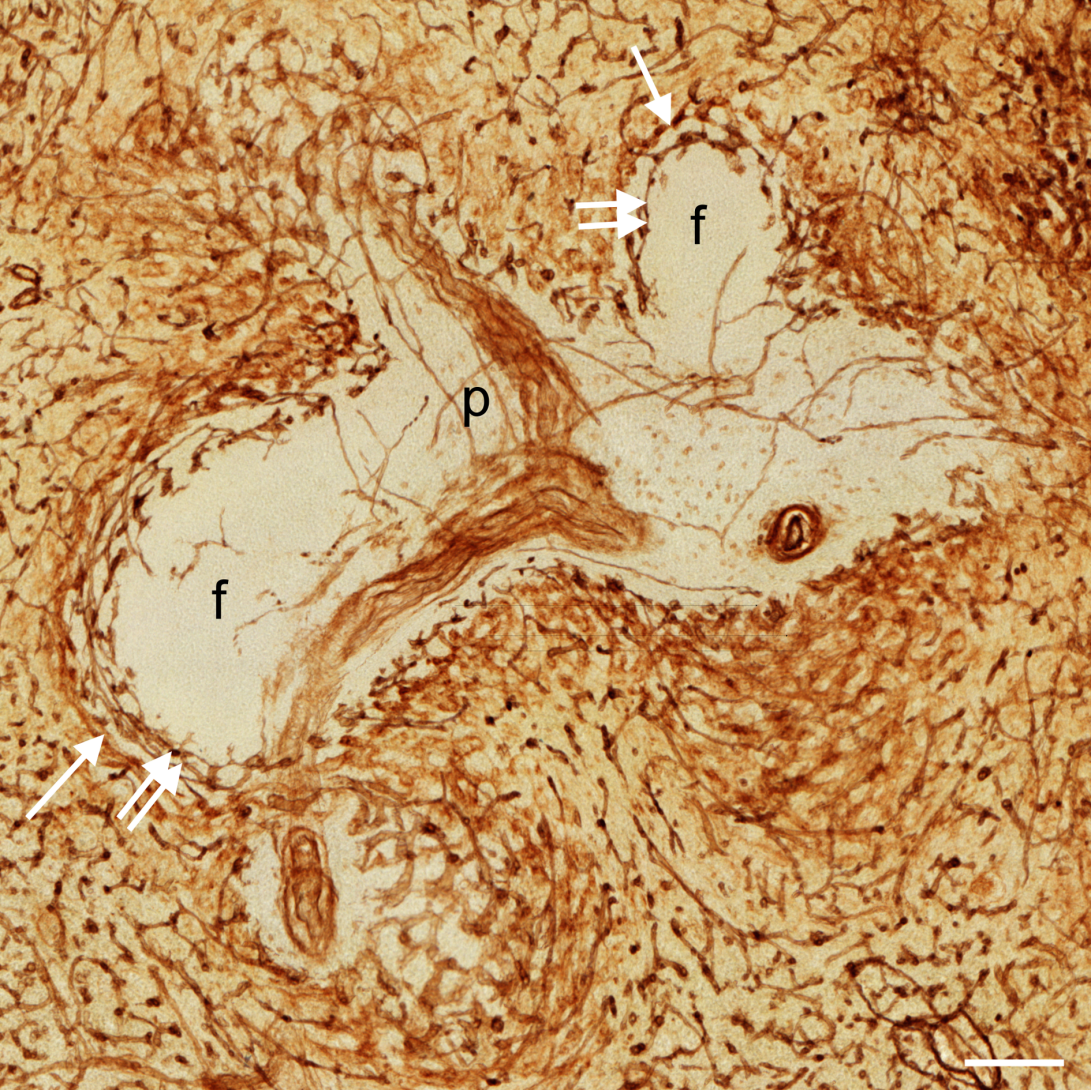
Visualisation of capillary networks centered on PALS and follicles. Distribution of CD34 in an overlay of 24 registered sections of ROI 4 showing a PALS (P) with a branching central artery and two sectioned follicles (f). There is a clear difference in the localisation of the perifollicular capillary network (double arrows) and the perifollicular sinus network (arrows). Internal capillaries of the PALS appear to run in the adventitia of the central artery forming vasa vasorum. Scale bar = 100 μm.

**Fig 4:**
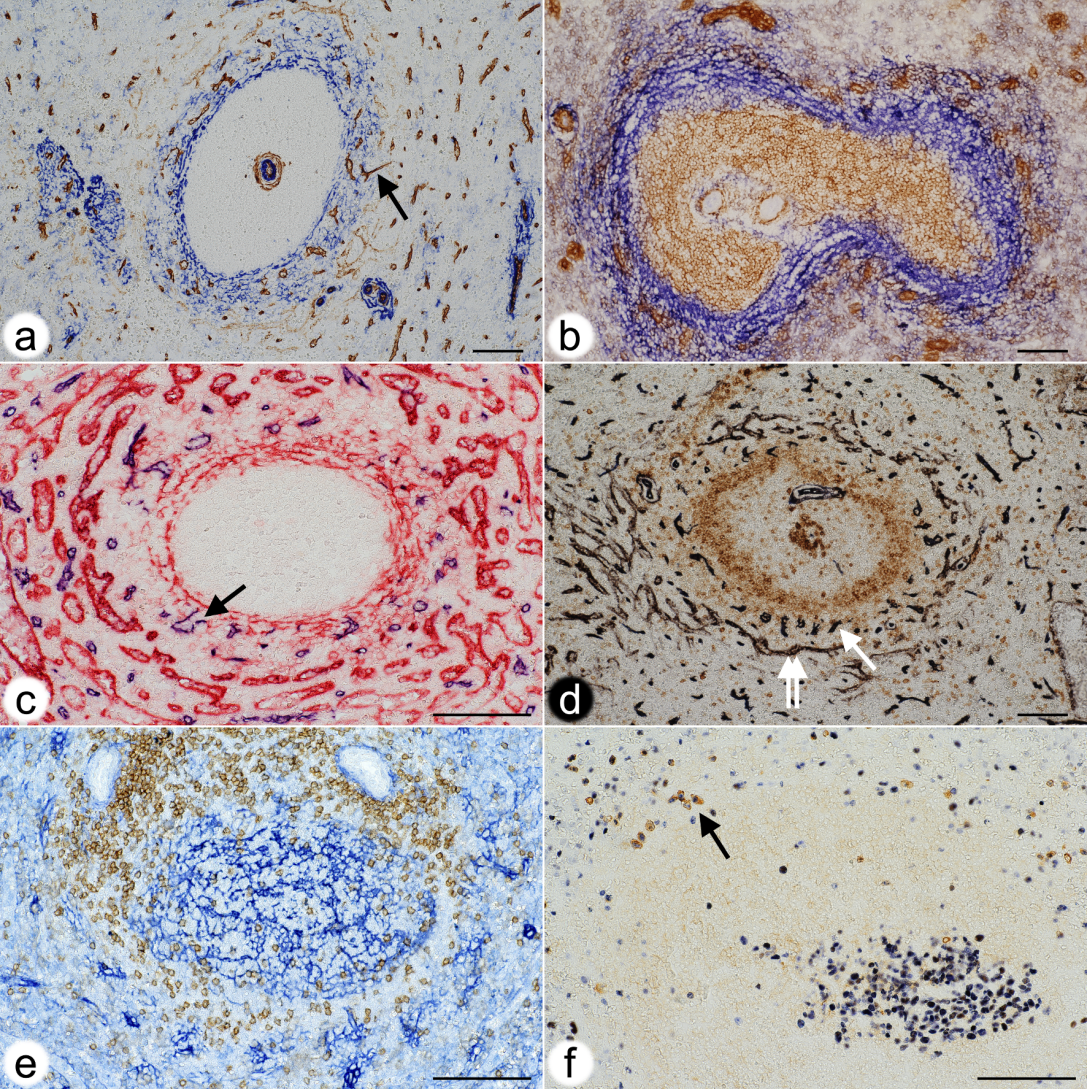
Localisation of the perifollicular capillary network in relation to stromal cells, B memory cells and plasmablasts. **(a)** Double staining for CD34 (brown) and SMA (blue) in a paraffin section. Strongly CD34^+^ capillary endothelia form a shell around the follicle. Sinus endothelia are weakly stained. A capillary (arrow) in the outer MZ forms a connection to red pulp. Same individual as Figs 1 and 2.**(b)** Double staining for CD271 (brown) and MAdCAM-1 (blue) in a cryosection. All FDCs are CD271^+^. MAdCAM-1^+^ perifollicular MRCs define the MZ and harbour capillaries surrounded by light brown fibroblasts. CD271^+^ capillary sheaths occur superficially in the MZ. Same individual as Figs 1 and 2.**(c)** Double staining for CD34 (blue) and CD141 (red) in a paraffin section. CD141 demonstrates venous sinuses in the red pulp and MRCs. Arrow indicates the open end of a perifollicular capillary. Same individual as Figs 1 and 2.**(d)** Double staining for CD34 (black) and CD27 (brown) in a paraffin section. The CD34^+^ capillary network is located in a superficial area of the follicle with scattered CD27^+^ B-cells. Double arrow shows perifollicular capillaries, arrow indicates sinus. Same individual as Figs 1 and 2.**(e)** Double staining for CD4^+^ T-cells (brown) and CD271^+^ FDCs and fibroblasts (blue) in a paraffin section. CD4^+^ T-cells originating from a PALS (upper part of image) occupy the CD271^-^ area underlying the perifollicular capillary network accompanied by CD271^+^ fibroblasts. Same individual as Figs 1 and 2.**(f)** Double staining for Ki-67 (blue) and intracellular IgM (brown) in a paraffin section. A germinal centre is located in the lower right part of the image. IgM^+^ plasmablasts with blue Ki-67^+^ nuclei (arrow) are located at the surface of the unstained follicle.Scale bar for all figures = 100 μm.

When single sections or overlays were inspected in the present investigation, large parts of the white pulp did not contain any CD34^+^ endothelial cells except in central arteries and their branches (Fig 1A, 1B, 1D, 1E, 1G and 1H, Fig 3). Few single faintly CD34 positive cells were seen in PALSs, which remain to be further analysed (Fig 1H, Fig 3). As described previously [4, 20], there was a network of capillaries in the red pulp. Careful analysis of the follicular surfaces for CD34 revealed that a network of strongly positive capillaries was located inside a ring of faintly stained larger sized sinuses (Fig 1A, 1B, 1D, 1E, 1G and 1H, Fig 3, Fig 4D). The individual perifollicular capillaries appeared more irregular and the network exhibited a different branching pattern than that found in red pulp capillaries (Fig 3). The perifollicular capillary network appeared to give off branches to red pulp capillaries (Fig 4A), which could not be followed further in single sections.

We first tried to localise the perifollicular CD34^+^ capillary endothelial cells in relation to the different cell types described to occur at the surface of human splenic follicles. The network was embedded in MRCs expressing MAdCAM-1, smooth muscle alpha-actin (SMA) and CD141 (Fig 4A–4C). In the specimen investigated most of the perifollicular CD27^+^ B-lymphocytes were located centrally to the perifollicular capillary network with scattered faintly CD27^+^ B-cells occurring between the capillaries (Fig 4D).

CD271, the low affinity nerve growth factor receptor p75, is most strongly expressed in capillary sheath cells of isoprismatic shape in the red pulp as well as in FDCs [14,18]. FRCs including adventitial cells around central arteries are less strongly stained. Fibroblasts at the surfaces of splenic trabeculae, but not in their interior, are also positive. In addition, there is a faintly positive ubiquitous stromal cell population in red pulp cords (Fig 2A, 2D and 2G).

These stromal cells appeared to express more CD271 in the vicinity of the perifollicular capillary network mentioned above (Fig 2A, 2B, 2D, 2E, 2G and 2H). The CD271^+^ fibroblasts surrounding the perifollicular capillary network were sometimes as strongly stained as capillary sheath cells, although they were morphologically different. Interestingly, there was a conspicuous CD271^-^ area at the follicular surface between the CD271^+^ FDCs and the CD271^+^ fibroblasts surrounding the capillary network (Fig 2A, 2D and 2G). This area continued around the PALSs. The CD271^-^ area around the follicles seemed to host those CD27^+^ memory B-cells (Fig 4D, [15]), which expressed CD27 most strongly. In addition, few scattered IgD^++^ cells, many CD4^+^ T-cells (Fig 4E) and—in one specimen—also a few plasmablasts positive for Ki-67 and intracellular IgM occurred in this location (Fig 4F). The CD271^-^ area was primarily supported by MAdCAM-1^+^ fibroblasts (Fig 4B; [14]) and might represent a special migration compartment. The innermost strongly CD27^+^ B-cells, however, clearly overlapped with typical CD271^+^ mantle zone FDCs [14]. Thus, CD27^+^ B-cells were found in two different stromal compartments defined either by FDCs or by MRCs at the follicular surface. This fact excludes that CD27^+^ B-cells can be used for defining a MZ equivalent in human spleens.

Detailed inspection of the perifollicular capillaries revealed that some of them had open ends (Fig 4C).

### 2.2 The perifollicular capillary network in 3D models

**Fig 5:**
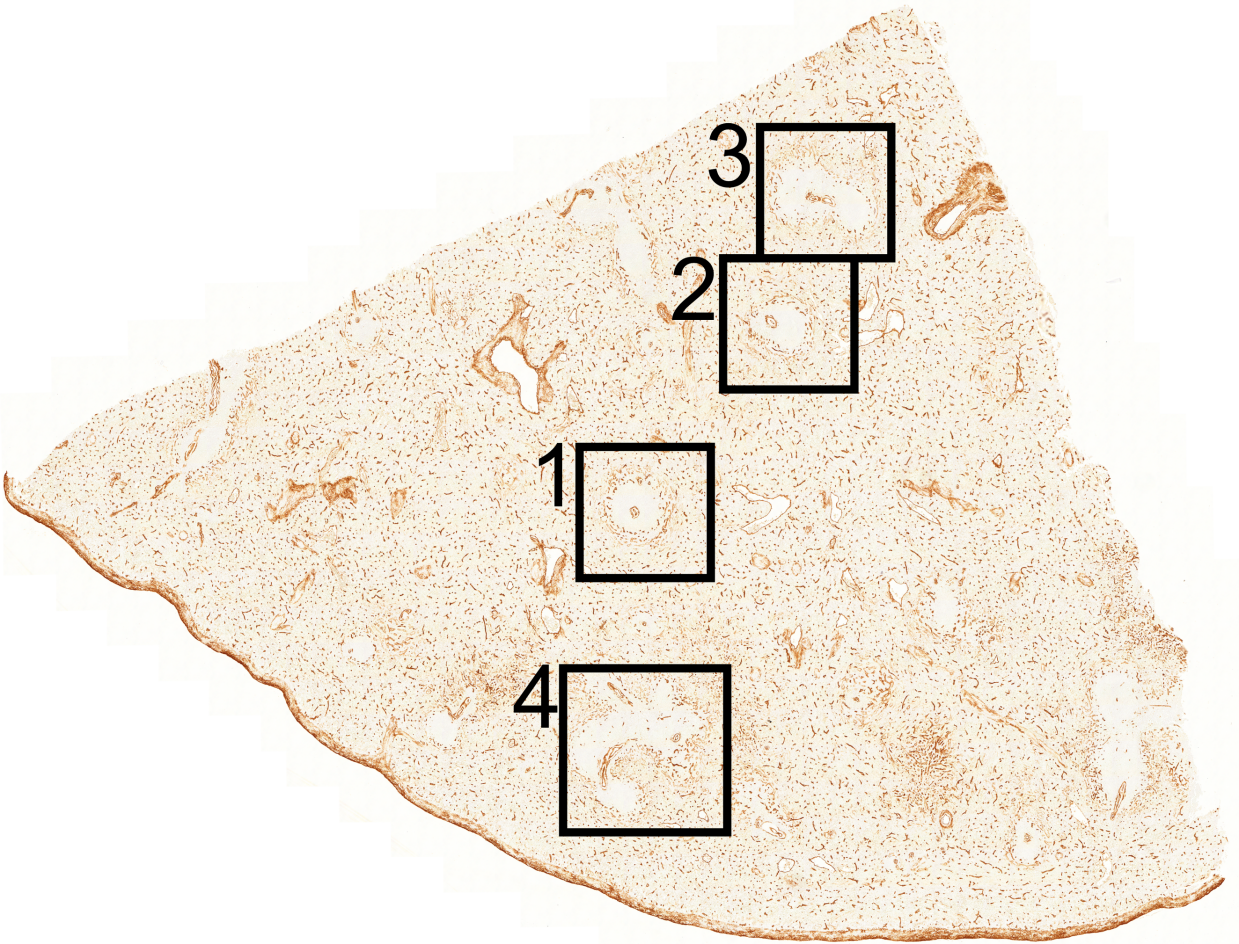
Overview of the section stained for CD34 showing localisation of ROI 1–4. The length of the cut edges is about 1 cm.

A 3D model of the perifollicular capillary network was constructed from 24 serial sections taken from a typical adult spleen. Four regions of interest (ROIs) were chosen for a detailed analysis (Fig 5). The serial sections were first stained for CD34 in brown colour. After scanning, they were stained for CD271 in blue and photodocumented again. Thus, ROI 1–3 were represented by a single-coloured and by a double-coloured data set. ROI 4 was only captured in brown. Both data sets were visualised as sequences of sections (S1a,b, S2a,b, S3a,b Videos at [https://doi.org/10.5281/zenodo.1039241]), overlays (Fig 1B, 1E and 1H; Fig 2B, 2E and 2H) and as 3D models (S1c,d, S2c,d, S3c,d Videos at [https://doi.org/10.5281/zenodo.1039241]). The single-coloured sets were used to visualise the perifollicular capillary network after optimal exclusion of the weakly CD34^+^ perifollicular sinuses. Exclusion of sinus staining was mandatory to find connections among the perifollicular and the red pulp capillary networks. Fig 1C, 1F and 1I and Videos S1c-S3c at [https://doi.org/10.5281/zenodo.1039241] demonstrate that the perifollicular capillary network is connected to the network of red pulp capillaries. The connections highlighted represent an underestimation of their real number, because of the rigorous filtering methods to exclude sinuses. It is highly likely that this procedure interrupted a large number of connections between perifollicular and red pulp capillaries.

Overlays of all sections (Fig 1B, 1E and 1H), sequences (S1a-S3a Videos at [https://doi.org/10.5281/zenodo.1039241]) and 3D models (Fig 1C, 1F and 1I; S1c-S3c Videos at [https://doi.org/10.5281/zenodo.1039241]) revealed astonishingly few capillaries inside the white pulp. Thus, the perifollicular capillary network appears to be primarily fed by red pulp microvessels. The follicles were located within their perifollicular capillaries similar to balls within basketball nets. The capillaries were clearly distinguishable from perifollicular sinuses (Fig 3). They appeared to continue at the surface of the PALSs, but this could not be investigated in detail because of the limited number of serial sections.

### 2.3 Perifollicular capillary sheaths in 3D models

Staining for CD34 in brown with subsequent visualisation of CD271 in blue revealed the arrangement of capillaries and capillary sheath cells in the surroundings of follicles. For 3D reconstruction, the iso-values for mesh construction were chosen to eliminate the ubiquitous light blue CD271^+^ fibroblasts in the red pulp, which would have obscured the model. Thus, we accepted losing the majority of the CD271^+^ FDCs and all FRCs to improve visualisation of capillary sheaths.

The frequency of capillary sheaths around follicles did not seem to differ from that in the red pulp. Interestingly, some of the perifollicular capillaries that had been demonstrated to be connected to the capillary network of the red pulp by single-staining for CD34 were shown to be connected to capillaries bearing sheaths (Fig 1C versus Fig 2C). Capillary sheaths were elongated and often branched structures of variable length. The CD271^+^ stromal sheath cells were isoprismatic and resembled epithelia. Their unstained nuclei often left gaps inside the cells, which sometimes provoked dissociation of sheaths during 3D reconstruction. The perifollicular capillary net was accompanied by branched CD271^+^ fibroblasts, which occasionally were so strongly stained that they could not be eliminated from the 3D model shown in Fig 2C, 2F and 2I and in S1d-S3d Videos at [https://doi.org/10.5281/zenodo.1039241]. These cells were, however, morphologically different from typical sheath cells.

### 2.4 Internal capillaries in the white pulp in 3D models

In two single follicles investigated (Fig 1A–1F; Fig 2A–2F; S1a-c at [https://doi.org/10.5281/zenodo.1039241], S2a-c Videos at [https://doi.org/10.5281/zenodo.1039241]) internal capillaries were not observed in 24 serial sections. To further analyse this phenomenon, we chose two additional regions, both containing two follicles and an associated PALS at low (Fig 1G–1I; Fig 2G–2I, S3a,c Videos at [https://doi.org/10.5281/zenodo.1039241]) and high magnification (Fig 3). In these regions it was evident that a few capillaries were present in the white pulp, which arose from side branches of the central arteries. The capillaries ran alone and had an extremely long and often rather straight course without branching. Some of these vessels appeared to form vasa vasorum for the central artery. Other capillaries, however, randomly traversed the white pulp without association with any histologically defined regions (Fig 1H and 1I; S3a,c Videos at [https://doi.org/10.5281/zenodo.1039241]). Interestingly, several of the internal white pulp capillaries seemed to end in the perifollicular capillary network (Fig 3).

## 3. Discussion

Our results show that three different but interconnected capillary networks occur in human spleens, a red pulp network, a perifollicular network and an internal network in the white pulp. It is likely that the perifollicular network is also present around the PALSs, but this was not investigated in detail. The red pulp network contains sheathed capillaries some of which are directly connected to perifollicular capillaries. We show that perifollicular capillaries form part of the open splenic circulation, because unequivocal open ends are observed in single sections.

We have chosen a sample of a representative adult spleen for investigation. Samples of this spleen have been immunostained with a large number of antibodies directed against different T- and B-lymphocyte populations, macrophages and stromal cells in the past [12–14]. We consider the organ as representative, because the findings obtained with these antibodies correspond to those seen in the majority of adult spleens with secondary follicles we have investigated during the last two decades. Up to now about 80 spleens have been studied altogether.

3D reconstruction is a tedious, time and data volume consuming process, which presently precludes investigating a larger number of individuals. A single scanned section (of 24) amounts to 2.4 GB in the original quality. Each single-stained ROI consisted of 4.1 x 10^8^ voxels after inter-slice interpolation, a double-stained ROI amounted to 8.5 x 10^8^ voxels. ROI 4 had 2.57 x 10^9^ voxels. The size of the initial meshes after the marching cubes procedure ranged from 345 to 1626 MB for brown staining. The total data of the 3D reconstruction project reached 517 GB. Because of the sheer data volume, the specimen and the ROIs for 3D reconstruction need to be very carefully selected.

Our investigation tries to solve the question whether there is an equivalent of the rodent MZ and the marginal sinus in human spleens. The answer is complicated by the fact that the MZ was first described in rats based on routine histology, which does not reveal all relevant cell types. In rats, it was first detected that the MZ is populated by memory B-lymphocytes [10]. In humans, the antigen most often used to detect memory B-lymphocytes is CD27, although this antigen is not present in all memory B-cells [16]. CD27^+^ B-cells can only be visualised after first staining T-cells, which also express this antigen [12,13]. Human CD27^+^ memory B-lymphocytes behave differently from rodent MZ B-cells. Most rat and mouse MZ B-cells are located superficial to all parts of the white pulp, i.e. to follicles, PALSs and the marginal sinus. The majority of human splenic CD27^+^ B-cells is, however, only found around follicles and is situated below the perifollicular capillary network, i.e. located towards the interior of the follicles. In apparent contrast to rodent MZ B-cells, human CD27^+^ B-cells appear in two different stromal compartments, characterised by CD271^+^ FDCs and by MAdCAM-1^+^ MRCs. Thus, the localisation of MZ B-cells appears to be species-specific. In rats and mice the MZ has long been regarded as a "static" compartment [9]. In contrast, it has also been reported, that rat and mouse MZ B-cells enter follicles in case of activation by LPS and other substances and perhaps even in the steady state [21–26]. Interestingly, mAbs detecting rat MZ B-cells also react with B-cells in follicles [27]. A final solution to this conundrum is still lacking.

3D reconstructions of serial sections have been attempted before [1,4,19,28,29]. Our registration method [30] is novel in the way it uses computer vision methods to better align the sections. In contrast, most other methods, such as Elastix [31], are image-based. Track-EM2 [32] from Fiji [33] also targets serial sections. The inter-slice interpolation we use [34] is a dense method. It utilises dense optical flow [35]. A sparse interpolation for electron microscopy has been suggested before [36]. Recently, a VR visualisation tool was published for medical applications [37], but we used our own custom-written VR visualisation software.

The monoclonal antibodies (mAbs) used are not totally specific to single cell types. This problem needed to be circumvented for 3D reconstruction. Thus, data processing of sections single-stained for CD34 was optimised to visualise only capillary and large vessel endothelial cells. We tried to eliminate the light brown staining of perifollicular sinuses by choosing an appropriate iso-value (S2a-d Figure at [https://doi.org/10.5281/zenodo.1039241]). This method inevitably led to the loss of connections among capillaries. The connections among the perifollicular and the red pulp capillary networks shown in Fig 1C, 1F and 1I and Videos S1c-3c at [https://doi.org/10.5281/zenodo.1039241] thus only represent examples of all connections which were present. The reconstructions visualise qualitative, but not quantitative facts. A similar condition also applies to the reconstructions of double-stained sections (Fig 2C, 2F and 2I; S1d-S3d Videos at [https://doi.org/10.5281/zenodo.1039241]), where the weakly CD271^+^ ubiquitous fibroblasts of the red pulp were eliminated to permit better recognition of capillary stromal sheath cells. This procedure was associated with the loss of a variably large number of CD271^+^ FDCs in follicles (Fig 2C, 2F and 2I; S1d-S3d Videos at [https://doi.org/10.5281/zenodo.1039241]).

Stringent quality control thus played a decisive role for interpretation of the 3D models. We achieved this by using a VR headset to walk through the reconstruction and inspect it from the interior and from all directions. VR permitted positioning each single registered section into the model so that all 3D structures could be directly compared with the staining results by viewing either the model (or parts of it) or the section or both (S4 Video at [https://doi.org/10.5281/zenodo.1039241]).

Up to now there is only one comparable investigation on the 3D structure of human spleen microvessels [1]. These authors used 50 to 100 serial sections to reveal CD34 and thus they were able to follow larger arterial vessels and their branches, which was not possible in our study. Somewhat similar to our results, Kusumi et al. did not detect capillaries in the human splenic white pulp and postulated that blood only comes from a superficial capillary plexus supplied by arterioles from the red pulp. Our results indicate that central arteries may have side branches supplying PALSs and follicles, but these vessels are astonishingly rare. We do not agree with Kusumi et al. [1] with respect to the location of the perifollicular capillary network as visualised in their schematic drawings. The authors postulate the existence of a MZ in human spleens without defining this region and they suppose that the capillary plexus exists at the inner border of the MZ. The analysis of cell phenotypes in our present and previous studies does, however, indicate that the CD34^+^ perifollicular capillaries are located within the area of MRCs located superficial to the majority of strongly CD27^+^ B-cells, i.e. they exist in the most superficial part of the MZ.

The scarcity of capillaries in the human splenic white pulp and especially in the follicles, may be related to the fact that adult human spleens are immunologically quiescent. Full-blown germinal centres seldom occur in healthy adults. If germinal centres are detected, they are rather small and non-polarised, although B-cell proliferation is still present (Fig 4F). Thus, spleens should also be investigated in immune reactions to see whether new white pulp capillaries appear on demand. However, the lack of internal capillaries may also be related to the necessity of maintaining a gradient of blood-borne mediators across follicles and PALSs to permit proper B-cell and T-cell recirculation. This may also be true for the access of antigen to follicles. In addition, the fact that most of the capillaries at the surface of follicles are supplied with blood from the red pulp might secure that the blood has previously passed sheathed capillaries. The function of capillary sheaths in humans is unknown. Many human sheaths—perhaps even all—are located in an immediate post-arteriolar position [15]. There are indications that immune complexes and carbon particles are retained in capillary sheaths termed ellipsoids in birds and other animals [38–40]. Our previous results have shown that human splenic capillary sheaths are composed of endothelia and pericytes, CXCL13^+^ stromal sheath cells, macrophages and recirculating B-lymphocytes [15,18]. Thus, sheaths may hypothetically extract large size antigens from the blood and participate in initial activation and guidance of naive B-cells immigrating into the spleen.

**Fig 6:**
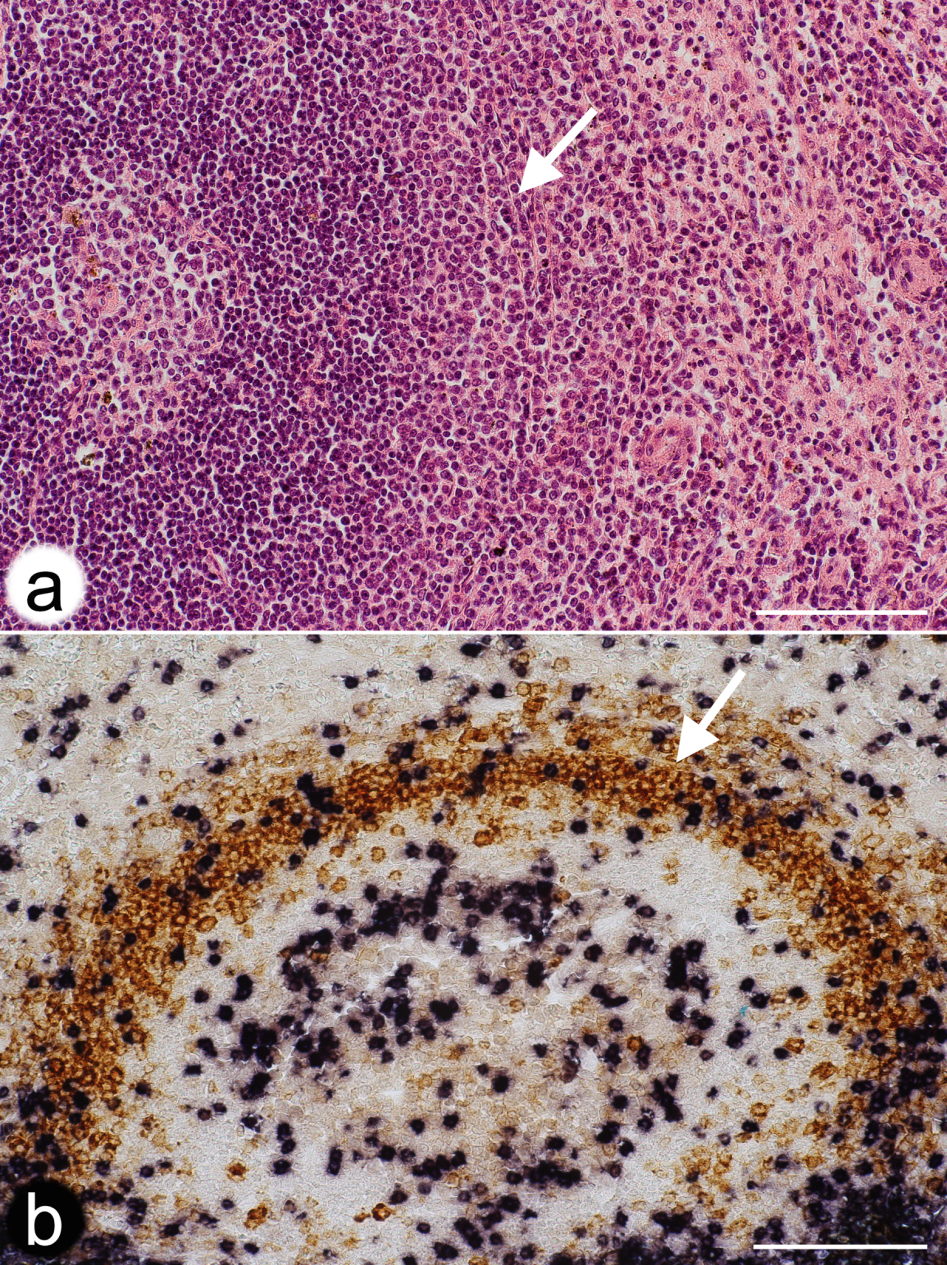
Localisation of a potential barrier towards the open splenic circulation at the follicular surface. **(a)** Hemalum-Eosin-stained paraffin section of a typical secondary follicle in an adult human spleen. Arrow indicates a layer of thick fibres and fibroblasts potentially forming a barrier against the open circulation. Same individual as Figs 1 and 2. **(b)** Double staining for CD3^+^ T-cells (blue-black) and CD27^+^ B-cells (brown) in a paraffin section. Arrow indicates fibres and fibroblasts outside the most strongly CD27^+^ B-cells potentially forming a barrier against the open circulation. Scale bars = 100 μm.

In mice and rats the marginal sinus does not only form the inner border of the MZ to provide direct access of blood to the MZ, but cells in its inner wall also establish a size barrier for substances approaching the white pulp from the open red pulp circulation [41]. Humans neither possess a marginal sinus nor marginal metallophilic macrophages. It is impossible to functionally investigate whether a barrier restricting size, charge or other properties of blood-borne substances also occurs in human spleens and which cell type is involved. The present investigation suggests that the innermost SMA^++^ MRCs (Fig 4A) might cooperate with MRCs of additional phenotypes to form a barrier between the open circulation of the red pulp and the white pulp. These fibroblasts and the thick fibres they produce are sometimes visible in conventional paraffin sections or in sections stained for CD27 (Fig 6A and 6B). They form the only barrier-like structure that exists at the follicular surface. SMA^+^ fibroblasts also occur in the inner wall of the marginal sinus in rats [42] and may also be responsible for establishing a barrier around the white pulp in this species. In humans, CD27^+^ memory B-cells and other lymphocytes may be easily able to cross such a barrier. Free erythrocytes are, however, only present outside the innermost SMA^++^ fibroblasts. Erythrocytes stay in an area, which may be termed the "outer MZ", which also harbours the perifollicular capillary network with surrounding weakly CD271^+^ fibroblasts. Switched IgM^-^IgD^-^CD27^+^ B-cells appear to also sojourn in the outer MZ [14]. This distribution may correspond to the distinct recirculation behaviour of non-switched and switched human memory B-cell populations noted by others [16]. Interestingly, the region immediately inside the putative barrier is a CD271^-^ region with strongly CD27^+^ B-cells. It may be termed the "inner MZ". The characteristic feature of both MZ compartments is the presence of MAdCAM-1^+^ stromal cells.

3D visualisation of capillaries combined with immunohistological staining for different cell types in 2D allows the following speculation: CD27^+^ B-cells in healthy adult humans enter the follicular surface from the red pulp in close association with MAdCAM-1^+^ perifollicular stromal cells by immigration from open-ended perifollicular capillaries or from the splenic cords. CD27^+^ B-cells then probe the CD271^+^ FDCs in the follicular mantle zone and leave again in direction to the red pulp if cognate antigen is not encountered. Probing may be especially important for non-switched CD27^+^ B-cells. This scenario is derived from the findings that—in contrast to IgD^++^ recirculating naive B-cells—CD27^+^ B-cells do not accumulate at any other place in human spleens. Only CD27^++^ plasmablasts and plasma cells, but not CD27^+^ B-cells, form groups of cells in the red pulp. CD27^+^ B-cells are also absent from capillary sheaths. Emigration back to the circulation most probably leads recirculating cells into the perifollicular sinuses. If the expression of CD34 in perifollicular sinuses is not a mere epiphenomenon, it may somehow help CD27^+^ B-cells and other lymphocytes to re-locate into the venous vasculature.

**Fig 7:**
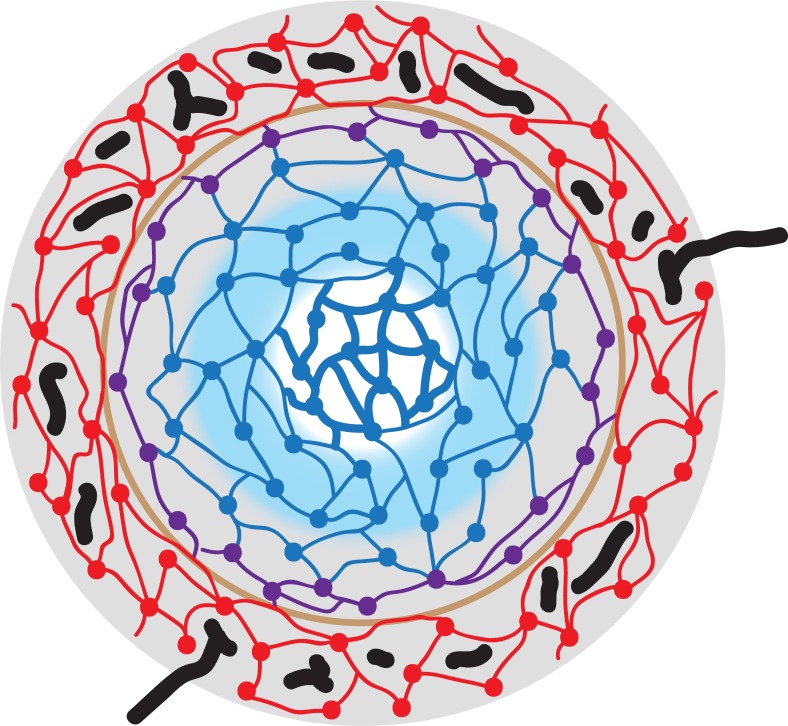
Schematic drawing visualising stromal cells, capillaries and the location of CD27^+^ B-lymphocytes at the surface of a secondary follicle in a human spleen. SMA^+^MAdCAM-1^+^ MRCs are depicted in red colour, SMA^-^MAdCAM-1^+^ MRCs in violet and CD271^+^ FDCs in blue. The innermost FDCs (thick blue lines) belong to the germinal centre. Perifollicular capillaries are black and the putative size barrier (formed by the red cells) is light brown. Gray background colour represents the area with CD27^+^ memory B-cells and blue background shows the follicular mantle zone. Note that the gray area overlaps the outermost blue FDCs and thus the mantle zone. There is no border between the mantle zone and the area of CD27^+^ B-cells. The follicle-associated T-cell area is not depicted.

The most strongly stained CD27^+^ B-cells at the follicular surface are located in a ring-like (2D) or shell-like (3D) region containing MAdCAM-1^+^ fibroblasts, but totally lacking any expression of CD271 in paraffin and cryosections [14,15], (Fig 7). This region also harbours CD4^+^ T-cells. In some spleens it is populated by a small number of Ki-67^+^cells containing intracellular IgM and coexpressing Ki-67, i.e. by plasmablasts, which continue at the surface of the PALS. These cells are supposed to be strongly CD27^+^ and may be partially responsible for the variable expression strength of CD27 in the inner MZ. It is likely that the CD271^-^ region represents a migration compartment, which is passed by cells moving either in parallel (plasmablasts and CD4^+^ T-cells) or at a rectangular direction (CD27^+^ B-cells) to the surface of the follicle. With exception of the innermost follicular CD27^+^ B-cells, MAdCAM-1^+^ fibroblasts and CD27^+^ B-cells are always closely associated at the follicular surface. It cannot be entirely excluded, that the expression of CD27 is upregulated in memory B-cells in the vicinity of follicles. This might explain the reduced staining intensity in the most superficial CD27^+^ B-cells. On the other hand, the cells may somehow be sorted for strength of CD27 expression by MRCs and FDCs.

Our present and past investigations [14,15] clearly show, that the human splenic marginal zone can only be defined by resident stromal cells, the MRCs. The most comprehensive phenotypical trait of human MRCs is the expression of MAdCAM-1, while SMA reveals a somewhat smaller MRC population [14]. Unfortunately, up to now human MAdCAM-1 cannot be reliably detected in paraffin sections, if cryostaining is used as a standard. Perifollicular MAdCAM-1^+^ fibroblasts have been found in each human spleen investigated so far, but these cells were lacking in tonsils (S1 Figure at [https://doi.org/10.5281/zenodo.1039241]). This is astonishing, because MAdCAM-1 is regarded as a typical adhesion molecule of mucosa-associated lymphatic tissues. Theoretically, it should be abundant in tonsils and absent from spleens, but in reality the reverse is true. Further antigens of MRCs, such as Notch ligands [43], remain to be reliably detected by immunohistology in humans.

In comparison to rats and mice, the surface of splenic follicles has a more complicated structure in humans, because an easily detectable barrier to the open splenic circulation is lacking. Blood is primarily transported to the follicles by a superficial network of fine capillaries located inside a network of large perifollicular/red pulp venous sinuses. This capillary network is part of the open splenic circulation and is primarily connected to red pulp capillaries. It may form an equivalent of the rodent marginal sinus, but its microanatomical location is different. If a size barrier for entry of blood-borne molecules into the white pulp exists in humans, it is most probably located internal to the capillary network and associated with MAdCAM-1^+^ and/or SMA^++^ MRCs. CD27^+^ memory B-lymphocytes occur in two different stromal compartments at the surface of follicles, defined by MRCs or by FDCs. Thus, only the perifollicular spleen-specific stromal cells, but not B-lymphocytes, define the human splenic MZ. A refined analysis of microvessels and stromal cells at the surface of mouse and rat splenic follicles is still lacking. Such a study is likely to reveal more similarities to humans than so far recognized.

## 4. Materials and methods

### 4.1 Specimens

The spleen specimen used for 3D reconstructions came from a 22-year-old male patient. It was fixed in 3,7% formaldehyde/tap water for 24 h, washed in tap water for 12h and processed to paraffin via graded solutions of isopropanol and xylol. Double staining in single sections involved this specimen and additionally a specimen of a 17-year-old male patient (Figs 4F and 6B). Both specimens were representative of a large number of adult spleens with small secondary follicles investigated during recent years. Human specimens were obtained before the year 2000. Acquisition conformed to the regulations of Marburg University Hospital at that time, when a formal ethics vote was not required. Verbal informed consent that a sample of the organ was to be used for basic anatomical research and was to be handed over to the first author was obtained from the patient by the attending surgeon in the Department of General Surgery of Marburg University Hospital. A hospital repository did and does not exist. The first author did not know the patients nor any data except age, sex and the fact that the patients had been healthy before splenectomy became necessary. Thus, the cause for splenectomy was unknown.

### 4.2 Single staining for CD34 in serial sections

The sections were de-paraffinised and stained for CD34 applying mAb QBend10 (Dianova, Hamburg, Germany No. DLN-09135) diluted 1: 1500 in PBS/1% BSA/0.1% NaN_3_ overnight after treating the sections with glucose oxidase (GO, Sigma-Aldrich Chemie Gmbh, Munich, Germany, No. G-6641) at 100 U/ml in PBS, pH 7.2, containing 20 mM beta-D-glucose and 2 mM NaN_3_) for 1 hr at 37° to remove endogenous peroxidase. Antigen retrieval was not performed. Binding of the mAb was visualised using the Vectastain Elite Kit for peroxidase (VEP, Vector Labs, Burlingame, USA, No. PK-6100) for mouse IgG (Vector Labs No. BA-9200) and diaminobenzidine (DAB) as chromogen. The sections were coverslipped in Eukitt.

### 4.3 Double-staining for CD34 and CD271 in serial sections

After scanning, the coverslips were removed in xylol, the sections were autoclaved in citrate buffer pH 6.0 and incubated with mAb EP1039Y (GeneTex No. GTX61425, via Biozol, Eching, Germany) overnight to reveal CD271. EP1039Y was diluted 1:150 and revealed with VEP for rabbit Ig G (Vector Labs, No. BA-1000). Bound antibody was revealed in blue using High Def Blue Peroxidase (HDBP, Enzo Life Sciences, Lörrach, Germany). The sections were coverslipped in polyvinyl alcohol (Mowiol).

### 4.4 Double staining for additional antigens in single sections

Unless indicated otherwise, immunohistology was performed in paraffin sections fixed in 3,7% formaldehyde for 24 h. With exception of double staining for CD34/SMA, where antigens were retrieved after visualisation of CD34, antigen retrieval was accomplished by autoclaving the deparaffinised sections in citrate buffer at pH 6.0 before the first antibody incubation. Endogenous peroxidase was always inactivated with glucose oxidase. Double staining for CD3 and CD27 was performed as described in [12]. Briefly, CD3 was revealed first using an alkaline phosphatase (AP) system and NBT/BCIP as blue/black chromogen, followed by staining for CD27 in brown using DAB. Double staining for CD271 and MAdCAM-1 had to be done in cryosections and has been described in [14]. Staining was first performed for CD271 in brown using DAB as chromogen followed by revealing MAdCAM-1 by an AP system and the Fast Blue chromogen. Double staining for CD34 and CD141 was performed according to [4] by first detecting CD34 in blue and subsequently CD141 in red using an AP system.

CD4 was detected by mAb 4B12 (DAKO, Hamburg, Germany, No. M7310) diluted 1:200 using VEP and DAB, followed by CD271 using mAb EP1039Y at 1:100 and the UltraVision System (LabVision, Fremont, USA via Thermo Fisher Scientific, Schwerte, Germany, No. TL-060-AL) for AP. AP was revealed by High Def Blue for AP (Enzo Life Sciences, Lörrach, Germany). For visualisation of CD34 and SMA, mAb QBend10 (Dianova, Hamburg, Germany, No. DLN-09135) was diluted 1:1500 and revealed with VEP (mouse) and DAB, followed by mAb asm-1 (Progen, Heidelberg, Germany, No. 61001) at 1:200 and the same detection system using HDBP as chromogen. For CD34 and CD27 mAb QBend10 was used at 1:1500 with VEP (mouse) and HDBP followed by mAb 137B4 (Quartett, Berlin, Germany, No. 030410901) at 1: 20, VEP (mouse), tyramide amplification and DAB. Ki-67 was shown using mAb MIB-5 (kindly donated by J Gerdes, Borstel, Germany) at 1:100 with VEP (mouse) and HDBP, combined with anti-IgM (DAKO, Hamburg, Germany, No. A425) diluted 1: 2000 for intracellular staining of plasma cells and DAB. All double-stained sections were mounted in Mowiol.

### 4.5 Image acquisition and processing for 3D reconstruction

#### 4.5.1 Image acquisition

The single-stained serial sections were acquired by VMscope GmbH (Berlin, Germany) with a Zeiss Mirax scanning microscope and a x20 lens. Double-stained ROIs of serial sections were acquired using a Canon 60D camera on a Zeiss Axiophot microscope with a x10 lens. The same camera was also used for documentation of all double-stained single sections.

#### 4.5.2 General outline of processing

The acquired data were normalised [44,45] and registered [30]. We defined ROIs that were processed and visualised. We show the resized registered sections as sequences (S1a,b-S3a,b Videos at [https://doi.org/10.5281/zenodo.1039241]) and as overlays, i.e. frontal volume renderings (Fig 1B, 1E and 1H; Fig 2B, 2E and 2H). We used inter-section interpolation [34] to reduce anisotropy. Colour processing reduced the data to a single channel. In case of double staining, the colours were separated and processed individually. We converted the volume data to a surface representation (a mesh) using the marching cubes algorithm [46]. The version we used included a simultaneous simplification [47]. The mesh was corrected for minor inconsistencies [48] and smoothed [49]. We also removed small unconnected components, typically those that were smaller than 2% of the main diagonal.

Our registration [30] uses a sparse approach. This approach matches and transforms detected image features instead of pixels and allows for processing of the whole sections [50]. Our registration features a non-rigid undistortion module to cope with individual distortions of the sections during cutting. This undistortion does not rely on a reference section. The module moves individual areas of the sections to minimise their distortion while maximising the number of features matched across the whole series.

In detail, we overlay a control point grid over the image. The control points are then moved driven by feature correspondence. The position of control points defines a spline-based undistortion applied to the image. We at once compute all undistortions in the series. Our registration is a multi-resolution method. After the first undistortion, the number of control points is doubled in each direction and the control points are moved again towards finer features, which results in a more refined correction of distortion. We typically use four iterations of our non-linear undistortion process during registration.

The inter-section interpolation [34] uses optical flow [35] to reduce anisotropy. Basically, further intermediate images are inserted between two sections. This provides for much smoother reconstructions. Using this interpolation the z resolution was improved from 7 μm to 1 μm.

We developed a special software for registration as well as for interpolation. Mesh generation was done with our own software for single staining data and with 3D Slicer (version 4.6.0, [51]) for double staining. Volume filtering for double staining was performed with 3D Slicer. We used Fiji (version 1.51n, [33]) for data conversion and for colour deconvolution. Mesh filtering was performed with MeshLab (version 1.33, [52]). Rendering was done with Cinema 4D (version R14.0429, MAXON Computer GmbH, Friedrichsdorf, Germany). The results are shown in Fig 1C, 1F and 1I and Fig 2C, 2F and 2I and in S1c,d-S3c,d Videos at [https://doi.org/10.5281/zenodo.1039241]. The models were quality-controlled in VR with our own specially designed software (S4 Video at [https://doi.org/10.5281/zenodo.1039241]). Videos were encoded with FFmpeg (version 3.2.2, https://ffmpeg.org).

#### 4.5.3 Single staining

For single-stained sections, we converted the registered stack to grayscale using Open CV [53]. The volume data were directly converted to a mesh without further mesh processing. This eliminated theoretically possible artefacts. Thus, some connections in the vasculature might have been lost, but artificial connections could be avoided during processing. For ROI 1–3 the data had 1600x1600x24 voxels at 0.6 μm/pixel in the x/y plane and 7 μm/section in direction of the z axis. ROI 4 had 4000x4000x24 voxels at 0.3 μm/pixel in the x/y plane and the same z resolution. The z resolution of all ROIs was increased afterwards to 1 μm/pixel by interpolation. Interpolation and mesh construction operated on grayscale images. The iso-value for ROI 1 and 2 was 119. An iso-value of 110 was used for ROI 3. The iso-value of 119 was initially optimised for best removal of weakly CD34-positive perifollicular sinuses (S2 Figure at [https://doi.org/10.5281/zenodo.1039241]). However, this value led to a lot of artefacts in ROI 3.

This problem may be due to slightly variable staining intensities of capillaries in different follicles caused by subtle variations in the qualitiy of fixation. Fixation may be better at the surface of the specimen and slightly reduced deeper inside the tissue.

#### 4.5.4 Double staining

3D reconstruction of double-stained sections required the separation of both staining colours and hence further processing. The brown staining of CD34 was separated as the black (K) channel of the image stack in the CMYK colour space. In our experience, this yielded best and most straightforward results compared to colour deconvolution. However, we had to use colour deconvolution [54,55] for the blue staining of CD271. It was not possible to obtain a reliable separation of the blue chromogen with simple colour space conversions, as was the case with the brown chromogen. Optical flow interpolation was performed after colour deconvolution. Before colour deconvolution, we removed background in Fiji [33].

Each channel had 2300x2300x24 voxels at 0.416 μm/pixel in the x/y plane and 7 μm/section in the z plane before interpolation. We interpolated to 1 μm/pixel in direction of the z axis, similar to other data sets.

The interpolated volume data were further processed before mesh construction. For the brown stain, we applied a grayscale closing filter with radius 7–7–3 and Gaussian blur with sigma = 1. This ensured smoother surfaces. For the blue stain, we changed the radius of the closing filter to 11–11–4. Otherwise, the processing was the same as for the brown stain.

After mesh construction for both brown and blue stains, we applied the same mesh processing steps as outlined above. An exception was the size of removed components for the blue stain, which was 10% of the main diagonal of the mesh. This action radically removed small unconnected structures in the reconstructions, while allowing us to maintain the sensitivity of a relatively low iso-value. The removal of small components was justified, as our objects of interest, the capillary sheaths, are rather large objects. Apart from capillary sheaths the most striking features of double staining for CD271 were FDCs in the follicles. The capillary sheath cells formed large connected structures in the reconstructions, which—in contrast to FDCs—were unaffected by the removal of small components. We also removed unconnected components smaller than 5% of the main diagonal for brown staining of ROI 1.

We typically used an iso-value of 80 for the construction of meshes from the volume data of both blue and brown stains. For the brown stain in ROI 1 we used a value of 130. The range of values in both cases is 0–255, as we used 8 bit per channel.

#### 4.5.5 Visualisation and quality control

The final meshes were rendered using Cinema 4D software. Minor processing improved the rendering of cut surfaces. We highlighted microvessels connecting the perifollicular capillary network with red pulp capillaries.Quality control of our reconstructions was performed using a custom-written VR tool. Hardware used for quality control included a HTC Vive headset and a PC with NVidia GTX 1070 graphics processing unit. Our tool allowed an intuitive inspection of the reconstructed meshes. It was also possible to visualise the image of the original registered section at the correct position in the reconstruction. This allowed us to inspect suspicious and/or interesting areas of the meshes with immediate reference to the original immunohistological staining (S4 Video at [https://doi.org/10.5281/zenodo.1039241]).
